# A machine learning pipeline for classification of cetacean echolocation clicks in large underwater acoustic datasets

**DOI:** 10.1371/journal.pcbi.1009613

**Published:** 2021-12-03

**Authors:** Kaitlin E. Frasier

**Affiliations:** Scripps Institution of Oceanography, University of California San Diego, La Jolla, California, United States of America; Cornell University, UNITED STATES

## Abstract

Machine learning algorithms, including recent advances in deep learning, are promising for tools for detection and classification of broadband high frequency signals in passive acoustic recordings. However, these methods are generally data-hungry and progress has been limited by challenges related to the lack of labeled datasets adequate for training and testing. Large quantities of known and as yet unidentified broadband signal types mingle in marine recordings, with variability introduced by acoustic propagation, source depths and orientations, and interacting signals. Manual classification of these datasets is unmanageable without an in-depth knowledge of the acoustic context of each recording location. A signal classification pipeline is presented which combines unsupervised and supervised learning phases with opportunities for expert oversight to label signals of interest. The method is illustrated with a case study using unsupervised clustering to identify five toothed whale echolocation click types and two anthropogenic signal categories. These categories are used to train a deep network to classify detected signals in either averaged time bins or as individual detections, in two independent datasets. Bin-level classification achieved higher overall precision (>99%) than click-level classification. However, click-level classification had the advantage of providing a label for every signal, and achieved higher overall recall, with overall precision from 92 to 94%. The results suggest that unsupervised learning is a viable solution for efficiently generating the large, representative training sets needed for applications of deep learning in passive acoustics.

## Author summary

Machine learning algorithms have proven to be effective for tools for detection and classification tasks in many fields, however, these processes are generally data-hungry and their use in marine acoustics has been limited by a lack of large labeled datasets for algorithms to learn from. In underwater acoustic recordings, many signals generated by animals, human activities and physical processes mingle together, and their sounds can change depending on ocean temperatures, locations, and behavior. Manual classification of these datasets is unmanageable without an in-depth knowledge of the acoustic context of each recording location. This paper presents a process which combines unsupervised and supervised learning phases and expert oversight to generate and use large datasets for acoustic classification of marine mammal and human-generated signals. Unsupervised learning is used to automatically generate the large training datasets needed to teach a supervised learning algorithm to correctly classify seven different signal types commonly recorded in the Southern California Bight. Using this process, researchers with large unlabeled acoustic datasets can begin to take advantage of widespread advances in machine learning.

This is a *PLOS Computational Biology* Methods paper.

## Introduction

Many high frequency, broadband, impulsive sounds are present in marine environments. Common sources include echolocating cetaceans, vessels, and echosounders. Passively recording these underwater sounds is an effective strategy for quantitative autonomous monitoring [1,2], however the recordings are acoustically complex and unlabeled. Moreover, advances in the longevity, sampling rates and storage capacity of passive acoustic recording technologies now facilitate the collection of extremely large datasets across widening frequency bands with high signal density, diversity and event overlap [3]. There is a growing need for detection and classification strategies capable of efficiently analyzing large, varied, unlabeled recording datasets for known and novel signals (e.g. [4]).

### Evolution of passive acoustic data analysis

Expert analysts have been relied upon to detect and classify events of interest in large acoustic datasets since the 1970s [5], typically by visually scanning through spectrograms and noting times of individual signals of interest or sets of co-occurring detections (often termed events, acoustic encounters, or bouts). Manual analysis is flexible, effective, and remains a key aspect of many recent passive acoustic monitoring studies [6–10]. Analysts are trained to recognize one or more signal categories of interest, and over time they gain an understanding of the within type variability of these signals, recognize related variants, and learn to minimize confusion related to environmental sounds and non-target signals [11]. For example, an analyst may become an expert in identifying and distinguishing regional beaked whale species, accounting for differences in noise, received level and orientation, and assigning any non-beaked whale signals to a generic “false” category [12–14]. This work requires careful attention to detail, and repeat scans through a dataset may be required if multiple signal categories are to be detected in this way. It is common to mark acoustic encounters or presence/absence of calls within a standard time interval rather than annotating individual signals [15], both for efficiency and broad statistical analyses, which may not require signal-level detail. However, these methods may limit more detailed interpretations of the data such efforts to infer species-specific densities, behavioral states, group sizes, or document mixed-species events. These types of analyses would benefit from more information on the quantity, rate of occurrence, acoustic characteristics and variability of detected signals. Manual labels vary in identifications and sensitivity analysts [11,16], and although analysts are highly-trained, the true acoustic sources are rarely known, and the best approximation of a ground-truth is taken to be consensus between multiple expert analysts (e.g. [17]).

Individually labeling echolocation clicks quickly becomes intractable in most monitoring applications, due to the large quantities of events. Automatic detectors have been developed to identify acoustic events that meet certain heuristics [18]. Classification steps often follow, designed to discard false detections associated with non-target sources and to further identify subcategories such as distinct species, signal variants, behaviors or orientation with respect to the recorder. These detectors and classifiers are typically tuned by expert analysts to detect particular species or signals of interest, based on their knowledge of the typical features of their target signal and the range of variability expected. Multiple detectors may be run, each tuned for a different signal type, and each attempting to reject different non-target signals.

### Machine learning: Promise with challenges

Machine learning methods have emerged as effective tools for classification in otherwise-intractable large passive acoustic datasets [19–21]. However, challenges related to production of labeled training data have limited advances hindered the adoption of promising deep learning methods, particularly for classification of high frequency broadband signals. Most marine mammals cannot be placed in a controlled environment for recording purposes, and even if they were, the complex, variable behaviors and ocean conditions that profoundly influence recorded signal characteristics would not be replicated. Major obstacles in labeled dataset development include poor understanding of the full range of possible classes, and an inability to develop ground truth label sets large enough for training and evaluating these often data hungry algorithms [22,23].

The classification task requires *a priori* knowledge of the expected classes, specific features associated with each signal class of interest, and the allowable variability of those features [24]. These classes must be defined, typically by an expert human analyst. As the quantity of detections, variability of datasets, and number of possible classes increase, the task of delineating classes and allowable ranges of within-type variability becomes more challenging. Acoustic datasets often include a mixture of well-defined and poorly-defined signal types. For example, sperm whales and some beaked whales produce well-characterized echolocation signals [25,26], while many delphinid species’ echolocation signals are only weakly parameterized, due to factors such as limited or overlapping spatial distributions, high signal variability, similarity between species, and limited signal information density. In addition, variation from canonical descriptions may occur across habitats, and the range of expected species and sources in a dataset may shift over time. Class definitions are further complicated by novel recording environments, noise, and transmission loss effects, as well as recording system differences [27].

The use of passive acoustic data for quantitative monitoring (e.g. densities, abundances, or any other unit intended to be comparable over time and between locations) requires estimation of classification accuracy for each target signal, which would typically be accomplished by comparing a representative subset of the classifications with a manually-verified ground truth [18,28]. However, with millions or more detectable impulsive signals across a large number of possible classes in a typical long-term acoustic recording, development of a manual ground truth becomes extremely difficult, time consuming, and potentially subjective. Recent advances toward density and abundance estimates using passive acoustic data using cue-counting methods rely on greater levels of label detail, either at the detection, short time window, or dive-start level [13,29–31], as well as the ability to tease apart overlapping events, such as a beaked whale encounter obscured by delphinids, or sperm whale echolocation mixed with ship noise. However, due to the short duration and minimal information contained in each detection, it is nearly impossible for analysts to make consistent detection-level determinations as to whether or not a signal has been correctly labeled. Contextual information, such cue rate and proximity of similar signals, is typically relied upon to make manual classification decisions [6], but context is lost when data are reduced to a set of independent automated detections. Conversely, too much reliance on context when designing automated classifiers can cause rarer signal types to be overlooked in cases where multiple signal types occur simultaneously. Recent studies have successfully used context to improve signal detection and reduce false positives, typically by increasing detector sensitivity during time periods with detections [32,33]. However, effective use of context in automated multi-species classification presents additional challenges related to defining probabilities of species and signal co-occurrence, particularly in high diversity settings.

Unsupervised learning used in combination with deep learning, and “human in the loop” review, may provide an approach for training and validating the next generation of acoustic signal classifiers. Rather than relying on analysts to define expected classes, unsupervised clustering algorithms can learn classes directly from one or more datasets of interest, disentangling within-type from between-type variability and determining the allowable distributions of signal features in each category. Prior work has demonstrated the use of unsupervised clustering to learn echolocation click classes across multiple acoustic monitoring sites [21]. In the present study, a workflow is described which integrates the unsupervised learning process with subsequent deep learning steps to train a classifier without the use of a manually developed training sets. This process can be used to automatically identify the major classes of impulsive biological and anthropogenic signals detected within one or more datasets, learn the characteristics of each class, and use learned characteristics to classify detections in a novel dataset.

## Methods

### Workflow description

**Fig 1:**
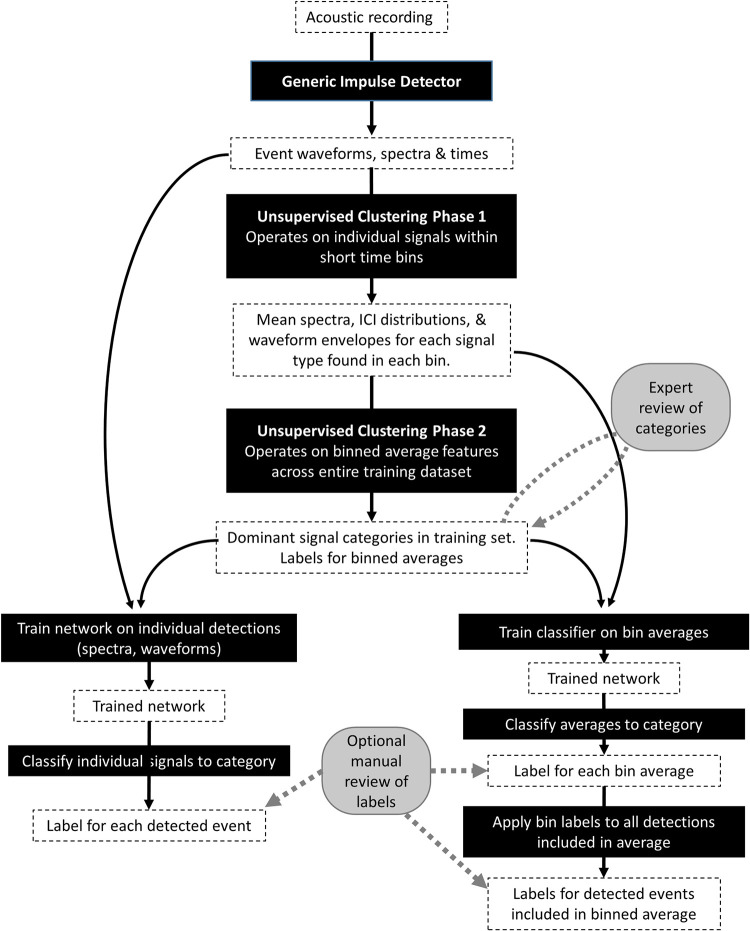
Flowchart describing the machine learning-supported workflow. White boxes indicate data inputs and produces, black boxes represent processing steps applied to the data. Gray circles indicate steps involving analyst review.

A description of machine-learning supported, human-in-the-loop detection and classification workflow follows (outlined in Fig 1). This workflow consists of 4 main steps: (1) generic impulse detection, (2) unsupervised clustering, (3) deep network training, and (4) classification of novel signals. The general design and intuition is described for each step, followed by the specific parameterization used in this case study. A detailed description of the study dataset concludes this section. All steps are implemented with user-interfaces within the publicly available acoustic data processing software package *Triton* [34] (github.com/MarineBioAcousticsRC/Triton), written in MATLAB [35], with the Signal Processing and Machine Learning Toolboxes. It can be run as described on a desktop personal computer with 32 GB of RAM with a 4-core CPU. An earlier version of this pipeline implemented in Python 2.7 using *Keras* [36] with *Tensorflow* [37] achieved similar results and performance.

### Generic impulse detection

In the first step of the workflow, a permissive, generic impulse detector is run. In this study, an energy detector implemented in Triton was configured to band-pass the data with a five-pole Butterworth filter from 5 to 100 kHz, and return signals with a received level ≥120 dB peak-to-peak re 1μPa^2^ and durations between 30 and 1200μs. Detections occurring within 100μs of one another were merged. (More information on detector specifics and implementation can be found in [21], and with implementation, source code and wiki available at github.com/MarineBioAcousticsRC/Triton).

The goal of this detector is to return all acoustic transients that exceed a static received level threshold and fall within very broad peak frequency and duration ranges. The rationale for this approach is that estimating false negative rates is more difficult than estimating false positive or misclassification rates [29]. False positive and misclassification rates can be estimated by manually reviewing a representative subset of detections. False negative rate estimation requires review of raw acoustic data to identify true events which met the intent of the imposed criteria but were missed. By erring on the side of detecting “everything” and then relying on algorithms to recognize the classes of signals, the detection and classification processes are less intertwined, and error rates may be more easily quantified. Additionally, this approach decreases processing times by reducing the need for multiple passes through the data with differently-tuned detectors.

**Fig 2:**
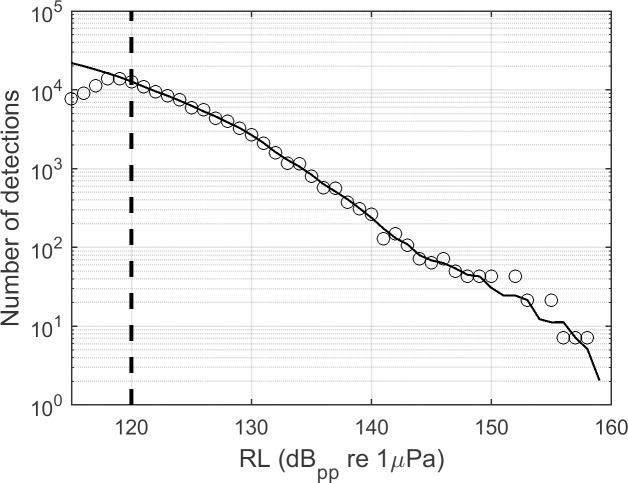
Modeled delphinid echolocation click received level distributions (solid black line) predict an exponential increase in the number of clicks detected (approximately linear in log space shown here) as received levels decline, assuming animals are uniformly distributed on average around a stationary sensor [38,39]. This shape is driven by an inverse relationship between range and received level (although signal directionality and other factors can introduce additional variation) and by the area monitored, which increases with the square of the monitoring radius, leading to greater numbers of animals at large ranges. Circles illustrate a typical “real-world” received level distribution from a click detector, in which detections approaching the intended threshold (115 dB_PP_ re 1μPa in this case) begin to be systematically missed. Enforcing a higher minimum amplitude threshold at which detection counts are still increasing (e.g. dashed line at 120 dB_PP_ re 1μPa) greatly simplifies subsequent analyses such as species-specific missed-rates and density estimates. More information on the model used in this illustration is available in [38].

The assumption that all target signals exceeding the minimum received level are detected can be checked by plotting the number of detected events as a function of received level (Fig 2), and verifying that the number of detections increases exponentially as the minimum received level threshold is approached [38]. Minor deviations from this relating to frequency dependent attenuation of different signals types, non-ideal bathymetry, or non-uniform source distributions may arise, particularly at higher received levels where sample sizes decrease. However, flattening or declining detection counts at the low end of the received level distribution is a clear indication that qualified events above the minimum received level threshold are being missed by the detector, typically due to masking. In these cases, if detector predictability is a priority in later steps, a simple solution may be to raise the minimum threshold to a level where signals are consistently detected.

#### Unsupervised identification of signal classes

The generic detector applied to a large passive acoustic dataset may return thousands to hundreds of thousands of qualifying detections per day, depending on the recording environment. Unsupervised clustering is used to identify consistent categories of signals within this set of events. The goal of this step is to develop a training set from which to train a classifier. A detailed treatment of this protocol can be found in Frasier et al. [21].

**Fig 3:**
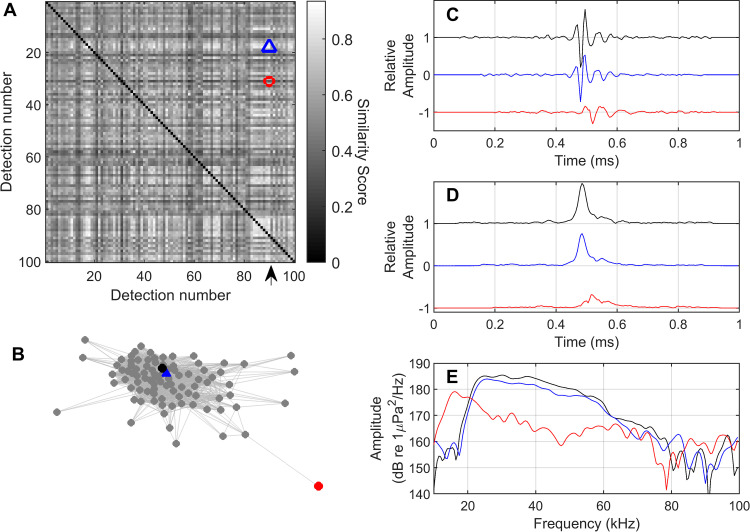
A similarity score based on correlation in time and frequency is used to associate similar signals and distinguish dissimilar signals. In this illustration, similarity between 100 detections is displayed as a symmetric similarity matrix (A). The similarity between detection X and detection Y is given by the color of grid cell (X,Y) on a scale between 0 (low similarity) and 1 (high similarity). Black squares along the diagonal represent comparisons of each detection to itself, and are ignored. The 90^th^ detection (a delphinid echolocation click) indicated by the black arrow in (A) is compared to two other detections: The blue triangle denotes a highly-similar detection, while the red square denotes a dissimilar detection. In (B) the same dataset is visualized as a network in which similar detections are attracted to each other and dissimilar detections repelled. The black node represents the 90^th^ detection. Waveforms (C), waveform envelopes (D) and spectra (E) are shown for the three detections, with the original detection in black, the similar detection in blue, and the dissimilar detection in red. Waveforms and waveform envelopes have been offset by a constant value for readability. Plots C-E indicate that the detections with high similarity scores are alike in the time and frequency domains, while detection with a low similarity score is quite different from the other two.

***Calculation of distances.*** To automatically identify the dominant signal types in a dataset, features of interest must be compared across a subset of detected events by computing a metric of similarity or “distance” calculated between each pair of events in the detection subset (Fig 3). The result can be imagined as a network in which each detection represents a “node”, and the distance between each pair of nodes is dictated by their degree of similarity. Highly-similar nodes cluster together, while highly dissimilar nodes are pushed far apart. For a fully connected set of N events in which a distance score is computed for every possible pair of events, the number of distances to compute is N^2^/2 (assuming a non-directional metric, i.e. that the distance between A and B is equivalent to that between B and A). This exponential relationship quickly makes subsequent computational analyses expensive and intractable for more than a few thousand detections. Furthermore, individual detections are short and highly variable, with limited information content. Rather than comparing millions of individual detections across an entire dataset, a two-pass approach is used to reduce the size and complexity of the dataset to be clustered, while amplifying and learning from the common features of similar detections which are proximal in time.

To generate distance metrics, signals are most easily compared in the frequency domain, avoiding the need to temporally align detected waveforms. If time domain comparison is desired, comparison can be performed using a signal envelope such as that estimated by a Hilbert transform, to minimize challenges related to phase alignment. Pairwise correlation distance [40] provides a computationally cheap metric for comparing shapes, and is less sensitive to amplitude differences and transmission loss effects than simpler metrics such as Euclidean distance.

Throughout the unsupervised learning process, the correct number of signal types is not known *a priori*, therefore clustering algorithms which do not require the number of categories to be specified are preferable. These algorithms, generally termed “agglomerative,” begin by assuming that each node belongs to its own cluster [41]. Nodes are then successively merged into common clusters following various strategies. The Chinese Whispers (CW) algorithm [42] is used in the described workflow. This approach iteratively assigns each node to the category to which it is most strongly connected, until reassignments cease or a maximum number of iterations is reached.

***Unsupervised clustering phase 1.*** It is assumed that in a short time interval (bin), detections will fall into a smaller number of classes than are present in the entire dataset, because the likelihood of observing multiple distinct signal classes decreases with the duration of the bin. Nonetheless, it is not uncommon to observe multiple signal types simultaneously. Unsupervised clustering is initially applied to sets of events detected in successive time bins to identify and summarize the signal type(s) in each bin.

Once similarities have been calculated between all nodes in a bin, edge pruning is used to reduce the size of the distance matrix input into the clustering algorithm. Only the highest 2 to 10% of similarity scores are retained for clustering purposes, with all weaker similarities remaining unspecified. This approach improves cluster formation but can result in exclusion of highly dissimilar events from any identified clusters [21]. Additional strategies for reducing network size include merging detections with extremely high similarity and selecting a random subset of detections when counts exceed a maximum threshold (10^4^ in this study).

Mean spectra, waveform envelopes and inter-detection interval (IDI) distributions are calculated for each bin-level cluster formed. A time bin may contain multiple clusters, which may represent different signal types, or variants of the same signal type. In some cases, disimilar and poor quality detections in sparsely-occupied time steps may fail to form a cluster.

***Unsupervised clustering phase 2.*** Bin-level averages are input into a second round of unsupervised clustering which operates on binned averages across the full range of the input dataset. On a desktop computer, 20 to 30 thousand bins approach an upper limit for simultaneous clustering in this phase. When inputs exceed this number of bins, a random subset may be selected. The set of binned clusters in this second round is generally limited to bins with a minimum number of events feeding into the average. A minimum threshold between 50 and 200 clicks per five minute time bin is used (an actively echolocating dolphin might produce one click every 0.06 ms, or 5000 clicks per 5-minute bin). Poor quality bins can be pruned from the final phase 2 clusters by removing a percentage of the most weakly-connected nodes from each cluster.

***Expert review.*** Upon completion of the second stage of clustering, time-binned averages have been partitioned into a set of signal types representative of the major categories observed in the input dataset. At this stage an experienced analyst can look at the signal types and assign them where possible to known sources (e.g. ship, Cuvier’s beaked whale, Risso’s dolphin) or otherwise label them (e.g. ‘unknown delphinid’). These labels can be propagated down to all of the individual signals contained in each reviewed cluster for detection-level learning.

#### Deep learning from identified signal categories

Once detections have been partitioned into categories by the unsupervised clustering workflow, the partitioned data can be used to train a conventional supervised classifier of the user’s choice, operating either on individual detections or binned averages. Deep neural networks lend themselves to classification based on entire signals and multiple inputs, minimizing the need for feature selection. For accurate classification, it is essential to retain both target and non-target classes in the training set [43]. Training, validation and test sets are formed from the input dataset.

**Fig 4:**
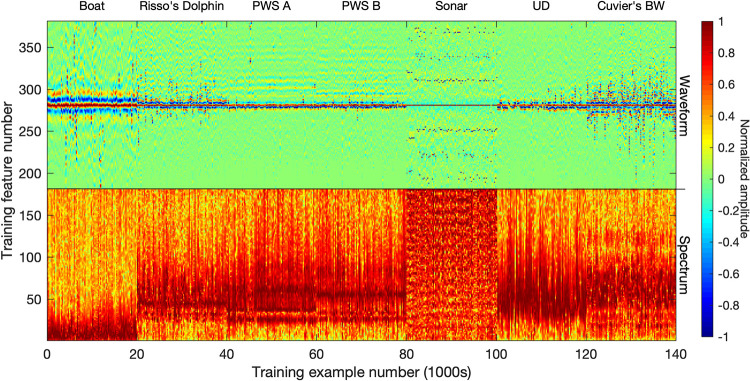
Input vectors for detection-level neural network training, consisting of concatenated spectra and waveforms of 140,000 detections (20,000 per class). Inputs are normalized as shown prior to being fed into the neural network.

***Individual detections.*** In the case of individual detections, the waveforms and spectra of individual clicks contributing to each of the mean spectra are extracted from each binned cluster. The entire time series snippet of a detection (1 ms duration) can be concatenated with the spectra to form the network training input (using 500 Hz frequency bins in this study; Fig 4). Alternatively, the input could be limited to either the time series or the spectra, or might include the time series envelope. Input spectra are standardized by subtracting a typical minimum value, and dividing by a maximum. Input waveforms are standardized by dividing by a typical maximum value. This approach retains some information about relative amplitude amongst detections, which can be useful features for classification, while reducing the range of observed values to a range better suited for network training (most values within the -1 to 1 range).

One advantage of classifying individual detections is that once the network is trained, it allows any novel input signal to be classified without a clustering step. In this case, clustering is only used for network training, and once trained, the network can simply classify new, similarly-standardized detections.

**Fig 5:**
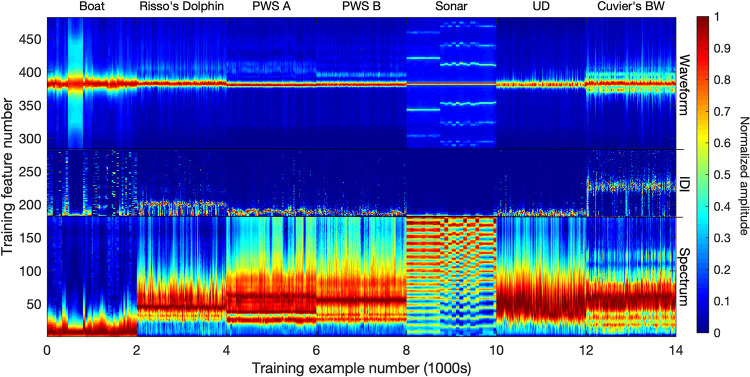
Input vectors for bin-level neural network training, consisting of concatenated mean spectra, IDI distributions, and mean waveforms of 14,000 bins (2,000 per class). Inputs are normalized as shown prior to being fed into the neural network.

***Binned averages.*** In the case of time binned cluster averages, the network inputs consist of concatenated mean spectra, mean waveform envelopes, and IDI distributions (Fig 5). By operating on clusters formed from multiple similar events co-occurring within short time intervals, this method includes some contextual information and the inclusion of IDI distribution as an input brings in temporal information not available on the scale of individual events. This additional information typically leads to higher classification accuracy; however, the resulting labels apply only to averages. The labels can be propagated to individual detections included in each average, however any detections which were not assigned to a phase 1 cluster cannot be classified with this method. These detections may include low quality detections dissimilar from other events in a time interval, events that were part of small, discarded clusters, and events that were excluded by subsampling in the case of too many events in a single time interval. This approach also requires novel input datasets to be processed through unsupervised clustering phase 1, to produce bin level averages, prior to classification.

***Network architecture.*** The underlying deep neural network structure is fundamentally the same for both individual detection and binned average options. Target labels are encoded as numbers, from 0 to N-1, where N is the number of target classes. Relatively simple deep network architectures are used, consisting of four 512-node fully-connected deep layers with 50% dropout between layers, leaky ReLU activations [44] and a softmax output layer [45]. Some signal types are far more common than others, therefore data may be subsampled and re-sampled as needed to create balanced training and testing sets (see discussion for implications of resampling).

#### Case study

**Fig 6:**
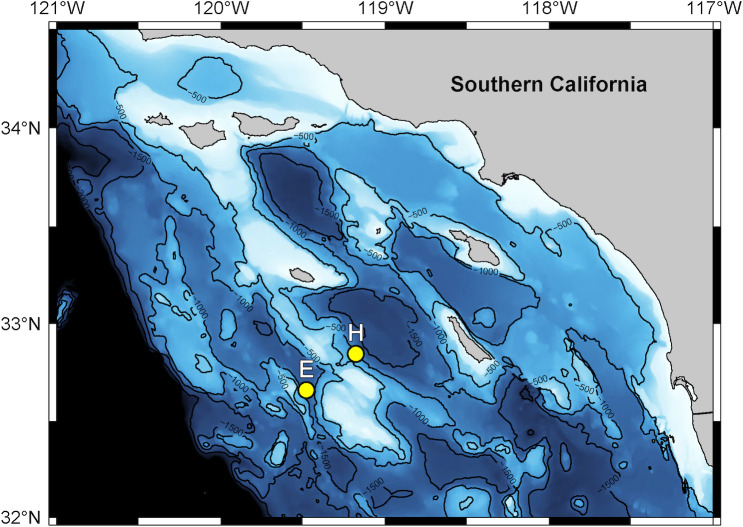
Map of HARP monitoring sites E and H, located in the Southern California Bight. Site depths are 1,300 m and 1,000 m respectively. Base map provided by [49] (https://www.bodc.ac.uk/data/open_download/gebco/gebco_2021_sub_ice_topo/zip/).

High frequency transient signals from sources including echolocation clicks from passing cetaceans as well as non-biological sources including boats and sonar were detected in two continuous passive acoustic recordings, collected at two distinct sites (E and H) in the southern California Bight in two different years (Fig 6 and Table 1; Data deposited in the Dryad repository: https://doi.org/10.6076/D1G01N; [46]) using High-frequency Acoustic Recording Package (HARPs; [47]). The unsupervised learning workflow was used to find the common signal types in the site H dataset, which were then used to train a deep network to classify the identified types. The classifier performance (precision, recall and confusion) was evaluated on the dataset collected at site E, by comparing the neural network labels to manual labels both at the five-minute bin level, and at the level of individual detections. Time bin-level labeling is a detection batching mechanism. Bin-level labels are applied to a cluster of similar detections, represented by one set of mean features or a distribution of feature values, occurring within some time window. Mean features tend to be more stable with lower noise than features of individual detections (Figs 4 vs. 5). Each bin-level label corresponds to a variable number of detections included in the mean or distribution. An alternative approach is to batch signals based on order, such as classifying the mean of 100 successive signals. A nice property of time-based binning over sequential batches is that it does not require an implementation of rules about how to handle gaps between encounters, and the bins can be related to density estimation problems in later steps. Detection-level labels are more intuitive, with a one-to-one match between labels and events.

**Table 1:** Deployment and detection information.

Site	Location (lat/lon)	Depth (meters)	Recording date range (mm/dd/yyyy)	Days of data	% Time bins containing > = 50 detections	Number of detections
H	32° 50.7’ N 119° 10.5’ W	1000	02/22/2017–06/06/2017	105	60.28	38,031,195
E	32° 50.5’ N 119° 10.2’ W	1300	11/28/2018–12/29/2018–01/25/2019–04/06/2019	122	55.68	38,127,204

The generic energy detector was used to detect signals with received levels ≥120 dB re 1μPa peak-to-peak. In Phase 1 of the unsupervised clustering step, detections were compared based on spectra. Phase 1 clusters were only retained if they contained at least 50 detections. In unsupervised clustering, bins were compared on mean spectra and waveform envelopes. Phase 2 clusters were only retained if they contained at least 50 nodes. An edge-pruning threshold of 90% was used in both phases 1 and 2. Phase 2 clustering was repeated 5 times and the best partition was selected as that which minimized the normalized mutual information criterion [21,48] and the most weakly connected 10% of nodes were removed to form the final set of clusters.

For each signal class in this study, events were split into encounters separated by a minimum of 15 minutes without detections of that class, and these encounters were randomly assigned to training, test or validation datasets, with 60% of the dataset used for network training, 10% for validation and 30% retained for testing. Two separate networks were trained, one on individual detections, the other on binned averages, to compare the strengths and limitations of the two approaches. To form the bin-level and detection-level training sets, 2,000 bins or 50,000 detections respectively were randomly selected from across the training encounters for each signal class. The evaluation sets were composed of 1000 bins, and 25,000 detections respectively for each signal class, randomly selected from the evaluation encounters. For classes with few examples, resampling was allowed to reach the required number of examples.

Networks were implemented in MATLAB using the Deep Learning Toolbox. In both the bin and detection-level cases, the deep networks consisted of an input layer, four 512-node fully-connected layers with 50% dropout between layers, and ending with a softmax output layer. Leaky Rectified Linear Unit activations were used [44] and the networks were trained with a constant learning rate of 0.0003 using a root mean square propagation optimizer. This simple design was used because it is extremely common and straightforward to implement in most neural network frameworks. A width of 512 was selected because it was the first power of two larger than the length of the input vectors.

Binned averages were normalized on a scale of 0–1 for network training purposes, while detections were standardized by subtracting a static low range value (70 dB re 1 μPa^2^/Hz) and dividing by a high range value (130 dB re 1 μPa^2^/Hz). Deep networks were trained with a batch size of 100 for bins, and 2000 for detections, with a patience of three training epochs, after which if performance on the validation set was not improving, training ceased. A maximum of 15 epochs were allowed.

## Results

### Detection

**Fig 7:**
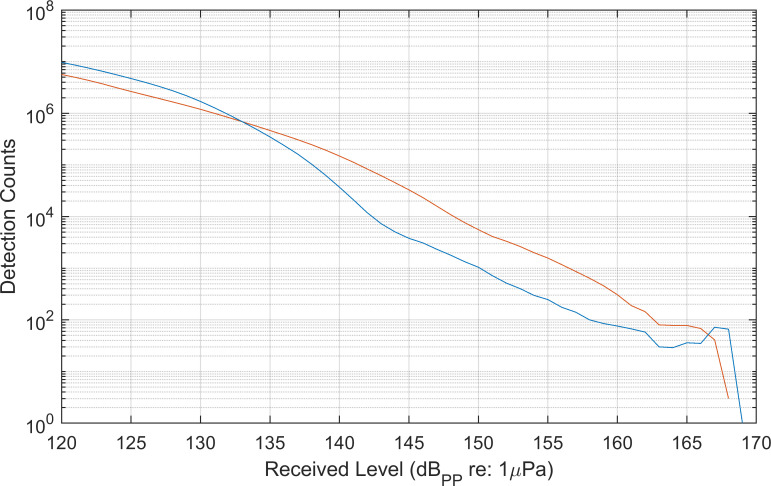
Received level distributions of detected signals above the minimum received level threshold of 120 dB_pp_ re 1 μPa. (Blue: Site E, Red: Site H).

Recording durations were 105 days at site H and 122 days at site E respectively (Table 1), with total approximately 38 million detections exceeding the minimum received level threshold at each site. Detection received levels were plotted to check for violation of the assumption that every detection which exceeds the minimum RL threshold was identified (Fig 7). Detection counts increased exponentially as the minimum detection threshold was approached, indicating that detections were not systematically missed near the detection threshold. The distributions of received levels differed between the two datasets, with larger numbers of higher received level detections occurring at site H.

### Unsupervised clustering

**Fig 8:**
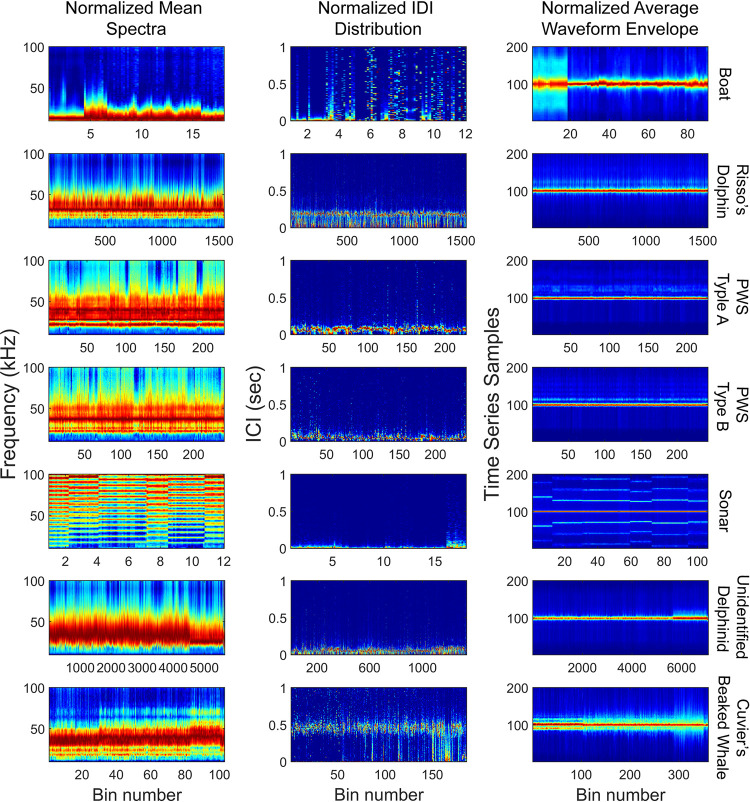
Signal classes formed from the training dataset using unsupervised clustering on spectra and waveform envelope. Seven signal classes were identified, including five odontocete echolocation types, ships and sonar. Color map represents normalized amplitudes on a scale of 0 (dark blue) to 1 (dark red).

The unsupervised clustering process was run on the site H detections. In the first phase, 19,301 bin-level clusters were identified, which formed seven signal type clusters in phase 2. Manual review of the clusters associated them with two anthropogenic sources (boats and sonar), and with five odontocete echolocation click types including: Risso’s dolpin (*Grampus griseus*), Pacific white-sided (*Lagenorhynchus obliquidens*; PWS) type A and B, Cuvier’s beaked whale (*Ziphius cavirostris*) and unidentified delphinids (UD) [28,50] (Fig 8; Table 2). Three distinct clusters were found in the expert review step to represent Cuvier’s beaked whale echolocation clicks, with the variations attributed to differences in transmission loss, and these were merged into one class. A large UD echolocation click category had considerable variability and may contain multiple, similar signal types associated with different dolphin species. Short-beaked common dolphins are the most abundant delphinid in the region and likely account for the majority of these detections, however long-beaked common dolphins, bottlenose dolphins, and occasional northern right whale dolphins are also present [51,52]. The majority of detections in the UD category are consistent with existing echolocation click descriptions for all of these species [28,53,54].

**Table 2:** Number of encounters from site H used for training (60% of available encounters), evaluation (30%) and validation (10%) of the neural network.

Signal categories	Training Encounters	Evaluation Encounters	Validation Encounters
Boat	16	8	2
Risso’s dolphin	182	92	30
PWS A	30	15	5
PWS B	49	24	8
Sonar	8	5	1
UD	491	247	81
Zc	111	56	18

### Classifier performance

#### Detection-level classification

Unique training examples ranged from 13,988 for sonar and 21,078 for boats to the maximum of 50,000 examples for the UD class. The detection level classifier completed 15 training epochs. The network achieved 92% classification overall accuracy on the balanced evaluation set from site E. Confusion was lowest on the more unique ship and sonar signals with near 100% correct classification (Table 3), and highest on the delphinids. Approximately 31% of examples labeled UD in the evaluation set were classified as other signal types by the network. Some confusion was observed between the two Pacific white-sided dolphin signals.

**Table 3:** Confusion matrix for click-level classifier on the balanced evaluation dataset from Site H, consisting of 25,000 examples per category. Values indicate percentages of the total number of detections classified.

		True Class							
**Predicted Class**		Boat	Risso’s	PWS A	PWS B	Sonar	UD	Cuvier’s BW	Predicted class precision
	Boat	**14.3**	0.0	0.0	0.0	0.0	0.0	0.0	100.0
	Risso’s	0.0	**11.8**	0.1	0.0	0.0	0.7	0.1	92.2
	PWS A	0.0	0.0	**12.9**	0.3	0.0	0.2	0.0	98.3
	PWS B	0.0	0.1	0.2	**12.2**	0.0	0.0	0.0	96.6
	Sonar	0.0	0.0	0.0	0.2	**14.3**	0.1	0.0	100.0
	UD	0.1	2.0	1.1	2.0	0.0	**13.3**	0.8	69.0
	Cuvier’s BW	0.0	0.3	0.0	0.0	0.0	0.0	**13.4**	97.2
	True class precision	100.0	82.5	90.0	85.1	100.0	93.1	93.5	92.0

**Fig 9:**
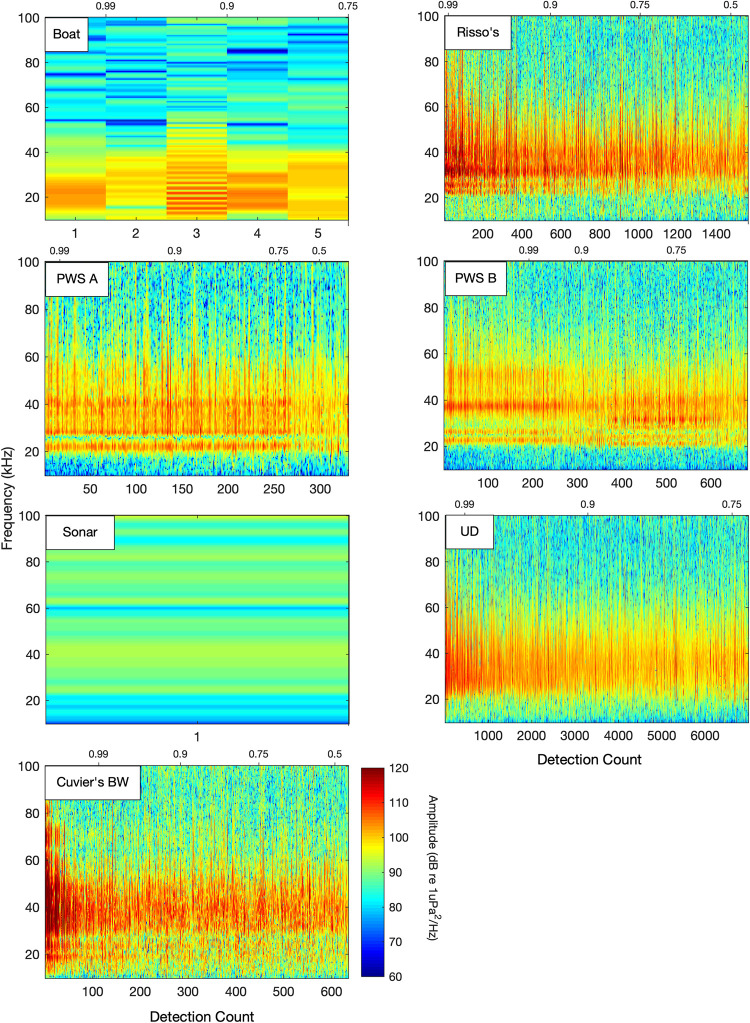
Spectra of detections counted as network misclassifications in the balanced Site H evaluation dataset. Spectra are sorted by classification probability scores shown along the upper edge of each subplot. A positive relationship between signal amplitude and probability scores is apparent, with higher amplitude signals being assigned higher probability labels by the network. Many of the signals counted as misclassifications appear to have been correctly classified by the network, but were likely incorrectly labeled in the unsupervised step used to create the training and evaluation datasets. For instance, the majority of spectra misclassified as Risso’s dolphin or PWS type A appear to have been correctly assigned to those species respectively.

Examination of the misclassified events revealed a mixture of signals which were misclassified by the network, and signals which were incorrectly labeled by the unsupervised clustering process used to generate the evaluation set (Fig 9). Classification probability was strongly related to signal amplitude, with higher label probability associated with higher amplitude signals. The majority of “misclassified” Risso’s dolphin, PWS Type A, UD and Cuvier’s beaked whale clicks appeared to be incorrectly labeled examples from the evaluation set which were assigned to their true category by the network. The high probability (> 0.9) PWS Type B detections also appeared to be accurately classified, but approximately 500 Risso’s dolphin detections were truly misclassified as PWS Type B. At lower received levels, detections of Cuvier’s beaked whale and Risso’s dolphin were sometimes confused, with lower confidence labels applied.

When applied to the unbalanced, manually labeled independent dataset from Site E, overall classification accuracy was slightly higher, at 94.2% (Table 4). In the Site E data, which represents typical class ratios for the Southern California region, 89.9% of the 38 million detections were manually labeled as UD, and 99.6% of those were correctly labeled by the classifier. However, due to the large numbers of delphinid detections, low misclassification rates still translated into fairly high numbers of misclassified signals.

**Table 4:** Detection level confusion on the independent, manually-labeled, unbalanced evaluation dataset from Site E. A total of 38,099,453 detections were classified. Detections are given in counts rather than percentages due to the large disparities between class sizes in this unbalanced dataset.

		True Class							
		Boat	Risso’s	PWS A	PWS B	Sonar	UD	Cuvier’s BW	Predicted class precision (%)
**Predicted Class**	Boat	**42,811**	220	2	1	35	149	213	98.57
	Risso’s	647	**647,902**	1,347	5,475	47	50,521	6,574	90.93
	PWS A	1,248	8,381	**708,005**	110,323	154	78,188	1,023	78.03
	PWS B	17	1,274	348	**4,562**	1	1,671	14	57.84
	Sonar	1,002	115	22	25	**72,924**	101	603	97.50
	UD	63,588	1,574,014	71,264	95,451	2,905	**34,079,189**	95,739	94.71
	Cuvier’s BW	695	17,200	152	211	352	3,492	**349,256**	94.05
	True class precision (%)	38.92	28.81	90.63	2.11	95.43	99.61	77.03	94.24

**Fig 10:**
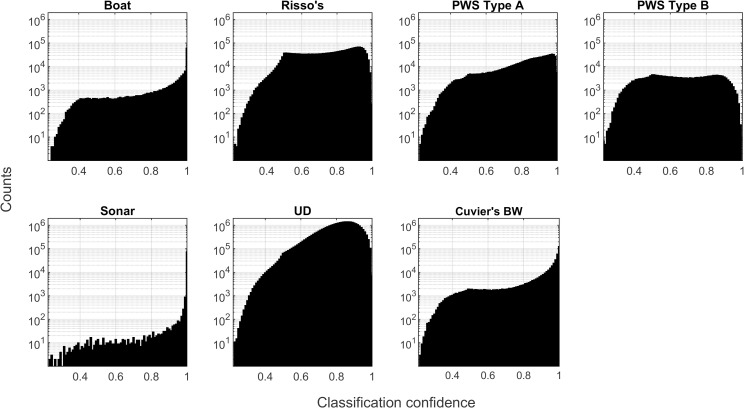
Distributions of detection-level label probability scores for each class for the Site E evaluation dataset. In this case, classes are taken to be those assigned by the network.

**Fig 11:**
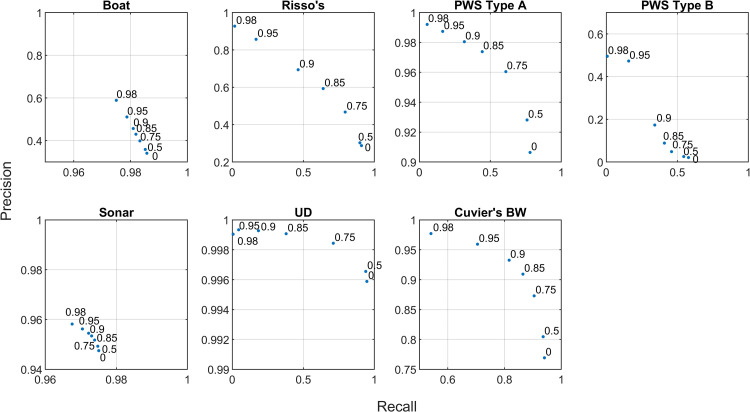
Detection level precision and recall curves for each class in the Site E evaluation dataset. Numbers within each plot represent thresholds applied in the classification probability scores assigned by the network, with points representing the precision and recall achieved by retaining only those labels with probabilities greater or equal to the associated threshold.

The distributions of classification prediction probability scores differed between classes (Fig 10). These probabilities do not represent a true, unbiased confidence metric, but are a metric of the network’s “confidence” on a scale of [0,1]. The boat, sonar, and Cuvier’s beaked whale categories included a large number of low probability labels, and a subset of very high probability labels. When the lower probability labels were discarded, precision increased considerably without severely affecting recall (Fig 11), suggesting that removing lower probability labels in these categories could further reduce confusion. The delphinid categories lacked this peak of very high probability labels, likely due to confusion created by imperfections in the training set and uncertainty associated with low amplitude detections. Very few PWS type B detections were present in the Site E dataset, and this contributed to low precision and recall for this class in the Site H data. Discarding low probability detections improved classifier precision for the delphinid classes, but the highest probability thresholds resulted in very low recall scores, suggesting that mid-range thresholds would more appropriate for these classes in this case.

#### Bin-level classification

**Fig 12:**
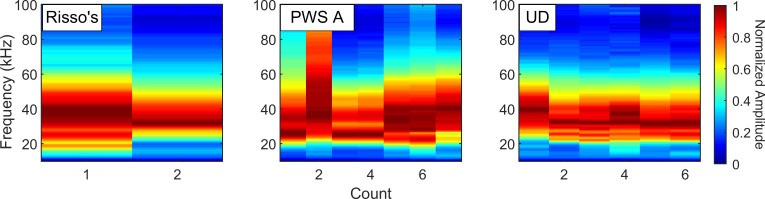
Spectra of bins counted as network misclassifications in the balanced Site H evaluation dataset. All detections had a probability greater than 0.9. Only classes which contained misclassifications are shown, a majority of the misclassified UD bins appear to be Risso’s dolphin bins. Some of the PWS Type A events are consistent with the class and may represent cases that were mislabeled in the cluster-derived Site H evaluation dataset.

Unique training examples ranged from 51 bins for boats and 24 bins for sonar to a maximum of 1,587 examples for the UD class. Network training ceased after 10 epochs as performance on the validation set ceased to increase. The network achieved 99.5% classification overall accuracy on the balanced evaluation set from Site E (Table 5). The largest number of misclassified bins appeared to be a subset of cases misclassified as UD which were likely Risso’s dolphin encounters (Fig 12). Some confusion was observed between the two pacific white-sided dolphin signals.

**Table 5:** Confusion matrix for bin-level classifier on the evaluation dataset, consisting of 1,000 examples per category. Values indicate percentages.

		True Class							
**Predicted Class**		Boat	Risso’s	PWS A	PWS B	Sonar	UD	Cuvier’s BW	Predicted class precision
	Boat	**14.3**	0.0	0.0	0.0	0.0	0.0	0.0	100.0
	Risso’s	0.0	**14.0**	0.0	0.0	0.0	0.0	0.1	98.9
	PWS A	0.0	0.0	**14.3**	0.0	0.0	0.1	0.0	99.1
	PWS B	0.0	0.0	0.2	**14.3**	0.0	0.0	0.0	100.0
	Sonar	0.0	0.0	0.0	0.0	**14.3**	0.0	0.0	100.0
	UD	0.0	0.2	0.0	0.0	0.0	**14.1**	0.0	98.3
	Cuvier’s BW	0.0	0.0	0.0	0.0	0.0	0.0	**14.1**	100.0
	True class precision	100.0	98.3	100.0	100.0	100.0	99.0	99.0	99.5

Classifier performance was lower on the unbalanced, manually labeled independent dataset from Site E. In this case, the network applied labels to one or more clusters identified within 5-minute time windows, while the ground-truth labels were applied at the detection level. For cases in which only one cluster was formed and labeled within the time window, but the manual labels identified multiple classes, a label of “none” was applied to account for the missing bin. Similarly, if no cluster formed, this was counted as a “none” label by the classifier for the purposes of computing accuracy metrics. These cases, which were not directly available to the classifier during the labeling stage due to clustering choices in prior steps, represented the majority of “misclassifications” in the bin-level labels (Table 6). True class precision was 81.2% including the “none” class, and 99.1% without it.

**Table 6:** Bin-level confusion on the independent, manually-labeled, unbalanced evaluation dataset from Site E. A total of 11,867 bin-level averages were classified. Bins are given in counts rather than percentages due to the large disparities between class sizes in this unbalanced dataset.

	True Class									
		Boat	Risso’s	PWS A	PWS B	Sonar	UD	Cuvier’s BW	None	Predicted class precision (%)
**Predicted Class**	Boat	**69**	0	0	0	1	6	0	0	90.8
	Risso’s	0	**485**	0	0	0	16	0	0	96.8
	PWS A	0	0	**911**	0	0	44	0	0	95.4
	PWS B	0	0	4	**10**	0	0	0	0	71.4
	Sonar	0	0	0	0	**55**	0	0	0	100
	UD	0	14	12	0	0	**8,598**	0	0	99.7
	Cuvier’s BW	0	1	0	0	0	4	**1,637**	0	99.7
	None	33	598	688	67	4	588	651	**0**	0
	True class precision (%) including “None”	67.6	44.2	56.4	13.0	91.7	92.9	71.5	0	81.2
	True class precision (%) without “None”	100	97.0	98.3	100	98.2	99.2	100	NA	99.1

**Fig 13:**
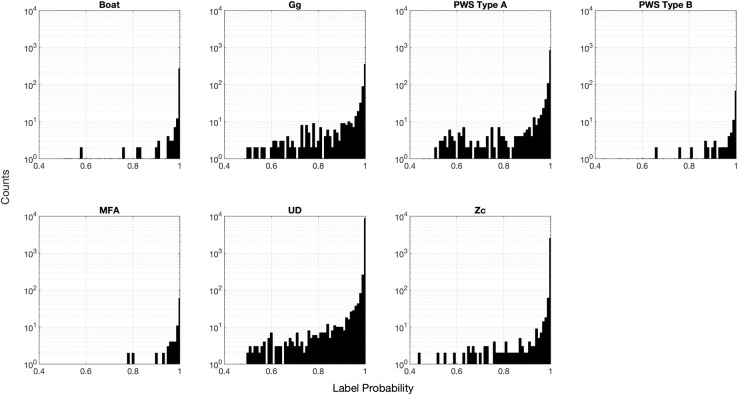
Distributions of bin-level label probability scores for each class for the Site E evaluation dataset. In this case, classes are taken to be those assigned by the network.

**Fig 14:**
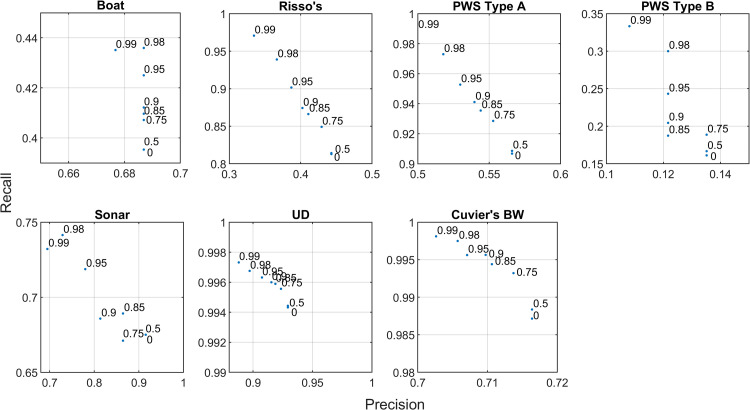
Bin level precision and recall curves for each class in the Site E evaluation dataset. Numbers within each plot represent thresholds applied in the classification probability scores assigned by the network, with points representing the precision and recall achieved by retaining only those labels with probabilities greater or equal to the associated threshold. “None” labels are included in these metrics.

The distributions of classification prediction probability scores were similar between classes in the bin-level case (Fig 13). Probabilities were heavily skewed toward high values, representing possible over-confidence by the network. Imposition of increasingly high probability thresholds did not result in large changes in precision and recall with the exception of the PWS type B class, which was rare in the Site E dataset (Fig 14). The weak relationship between probability scores and improved accuracy suggests that this metric may not be particularly useful in the bin-level case.

**Fig 15:**
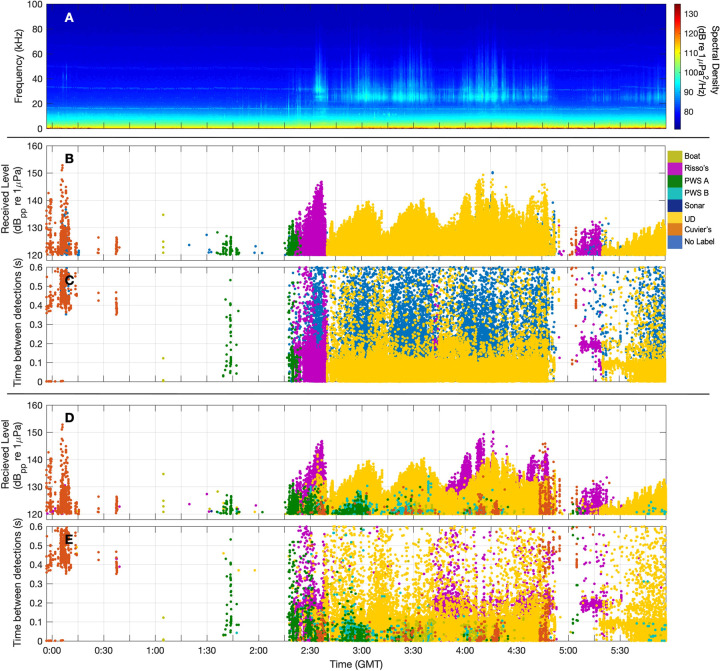
Comparison of detection and bin level classification results on a six-hour data segment from Site E, displayed as long term spectral average (LTSA) (A). Classes assigned by the bin-level network are shown in (B) as a time series of detection received levels, and in (C) as a time series of inter-detection intervals. Each point represents one detection, and color indicates class assigned by the bin-based classifier. Blue points represent detections which were not assigned a class. Classes assigned by the click-level network are shown in (D) and (E). Note that every click is labeled in the click-level case.

A comparison of automatic labels applied by the detection-level and bin-level labels is shown in Fig 15. The detection level classifier applied labels to every detection, but low levels of probable misclassifications as a mix of colors overlayed on the UD category. In this example, the detection level approach is more effective at identifying temporally overlapping encounters with different species, such as Risso’s dolphin and the UD category. In contrast, the bin-level network reduces the occurrence of spurious, low probability mixed species labels. Using this approach low quality echolocation clicks are often left unlabeled.

## Discussion

As passive acoustic datasets increase in bandwidth and duration, manual review becomes less practical. However, without manual review, we risk finding and quantifying only the signal categories that we know to look for, with distributions dictated by past observations, thereby limiting novel discoveries. These limitations are problematic in the rapidly developing field of passive acoustic monitoring. The described workflow facilitates discovery and learning of both known and unknown signals within large recording datasets, without requiring *a priori* knowledge of the data, or expert parameterization of target signals of interest. Unlike most existing detection strategies which are tuned for specific target signals, this method is flexible and broad, with the potential to reduce time spent running different detectors for different target signals (audio file I/O is a critical bottleneck in long term acoustic data processing). The unsupervised portion of the algorithm is able to adapt to site, region, or season-specific variation in signal frequency content and production rate.

The unsupervised clustering process does require basic decisions regarding which features to compare and eventually train classifiers on. Options include waveforms or rectified waveform envelopes, spectra, and IDI distributions. During testing, waveform and spectra produced the best output clusters, with fewest duplicate clusters and minimal mingling of similar types. IDI was often a characteristic feature, however its use in the unsupervised clustering phase tended to produce duplicate clusters with similar spectra but slightly different timing. In this case study, although IDI was not used for clustering, clear modal IDI patterns emerged in the Phase 2 clusters nonetheless, emphasizing that IDI is a useful feature for classification. For that reason, it was used as an input when training bin-level neural network classifier. Timing has proven less useful for classification at the level of individual detections. Time to the next detection was tested with minimal success, presumably because it is highly variable and easily modified by group size or overlap during mixed species events.

The dataset used here is collected in deep water with near-seafloor sensors, therefore, shallow diving species (e.g. dolphins) are likely to be further from the sensor than they would be in shallow environments. Directional echolocation clicks are less likely to be received off-axis in this case because their received levels with respect to the sensor are much lower than those of on- or nearly on-axis signals. This reduces overlap between recorded click trains at deeper recording locations relative to near-surface or shallow water recordings, and allows modal IDI patterns to appear more readily (although when groups are large or nearby, modal IDIs typically disappear). IDIs may be less diagnostic in shallow water (including towed array data) due to overlapping click trains, reflections, as well as the introduction of unexpected signals like snapping shrimp or rain. Additionally, background noise, signal amplitudes, and signal to noise ratios vary across recording environments and can affect classifier performance. For these reasons, it is important to select a training set that is representative of the data intended for classification.

The unsupervised clustering approach does not ensure the creation of perfect training and evaluation sets, particularly in the detection level case. This is illustrated in this case study by the numerous “misclassified” detections which appeared to actually correctly categorized by the classifier, but mislabeled in the ground truth. In the detection-level case, these misclassifications arise when multiple signal types are detected simultaneously, such as when multiple acoustically active species are within range of the recorder, or ship or echosounder signals are mixed with a marine mammal echolocation, and are not well separated in the Phase 1 clustering step, leading to bin-level averages which contain multiple click types. Additional cleanup steps could be implemented to improve the purity of the training and evaluation sets generated with this process, which would likely improve performance further by reducing confusion.

One weakness of the unsupervised approach is a somewhat limited ability to recognize rare signal types as distinct categories. A number of species occasionally present in the Southern California Bight such as sperm whales, Baird’s beaked whale and *Kogia* spp. would not be recognized by the classifier presented here because they were not detected in large enough numbers in the recording used for training. In practice, the simplest solution for this is to augment the training dataset with classes from datasets collected during other seasons or at other locations. Clusters could be brought together from across a range of sites of interest to form the neural network training set, expanding the number of learned signals, and improving handling of different recording conditions, background noise, or other sources of variability across sites, seasons and sensors. Care is required to ensure that combined training sets are structured such that instrument self -noise or background noise features are not being learned rather than the target signals. Small classes in the training could also be augmented set by resampling and adding noise, or by re-combining observations to produce slight variations. However, these approaches negatively affected performance on real data in testing, and more development is required to implement these methods effectively. Random resampling of observations was allowed in this study to achieve balanced training and evaluation sets, however heavy resampling can result in brittle class definitions, and a weaker ability of the network to recognize variants of rare signals when applied to a novel dataset. This may explain the high confusion associated with the relatively sparse PWS type B class using both detection and bin-level methods. Performance on that class would likely be improved by augmenting the training set with additional examples from other deployments. Misclassification of rare species is an important issue, as it can lead to significant errors in later steps such as density estimation [55]. Manual review and editing of the labels using a batch review tool such as *PAMGuard* [56] or *DetEdit* [6] remains particularly important in these cases.

In addition to adequate representation of target classes, it is important to ensure that the major non-target categories are also represented in the neural network training set as out groups. Including a non-target class in the training set generally reduces spurious assignment of unrepresented non-target signals to target categories.

It is worth noting that waveforms contain the information provided in the spectra, therefore for classification purposes, the two could be considered redundant. However, when operating on waveforms alone, the classifier was more sensitive to differences in amplitude and background noise, resulting in notable increases in confusion between high amplitude delphinid and low amplitude beaked whale signals. Furthermore, for bin level labeling, waveforms have to be averaged, which requires accurate time alignment. Precise time alignment is computationally expensive for this number of detections, therefore it was preferable to compute an average waveform envelope, which is less sensitive to phase, and to include mean spectra as the classifier feature set.

### Future work

Expert review is still required after the unsupervised clustering phase to identify duplicate clusters and verify that closely-related signal types such as distinct dolphin species are separating correctly. There is ample opportunity to investigate alternative unsupervised clustering methods which may better distinguish between closely-related signals (e.g. different types of dolphins), without splitting up variations of the same signal into multiple clusters (e.g. beaked whale signals under different transmission loss). To date, we have tested a few clustering methods including modularity [57], decision trees, Louvain [58], DBSCAN [59] and a range of distance metrics, with less desirable results, however other algorithms or combinations are likely possible.

These results clearly show potential of click-level labeling, however classification accuracy of individual signals with low classification certainty could likely be improved by considering neighboring signals to provide additional context. For instance, if many neighboring signals are classified as Cuvier’s beaked whale then the odds that a poor quality in their midst is also Cuvier’s beaked whale is probably higher. Although this approach could reduce low level random misclassifications, this type of decision making must be applied carefully to avoid missing small events such as a brief beaked whale encounter embedded within a multi-hour dolphin encounter. A possible approach may be to add a second classification network capable of adjusting labels based on neighbors, or to merge bin-level and detection level classifications. Multiple networks trained on different subsets detections could be ensembled to obtain estimates of label uncertainty.

## Conclusion

The proposed signal classification pipeline combines unsupervised and supervised learning phases with opportunities for expert oversight to label signals of interest, some of which are fairly well known, such as certain beaked whale species, and others which are not yet well characterized or matched to a known source. The method is flexible to facilitate learning signals from novel recordings and can be adapted according to the level of signal specificity desired by the user. Bin-level classification was found to achieve higher overall precision than click-level classification due to additional IDI information and clearer signal features achieved by averaging many signals. However, click-level classification had the advantage of providing a label for every signal, and achieved higher overall recall. The results suggest that unsupervised learning may be a viable solution for efficiently generating the large, representative training sets needed for applications of deep learning in passive acoustics.
